# Crisis social support after work-related violence and threats and risk for depressive symptoms: a 3-months follow-up study

**DOI:** 10.1186/s40359-023-01081-x

**Published:** 2023-02-11

**Authors:** Lars Peter Andersen, Ask Elklit, Jesper Pihl-Thingvad

**Affiliations:** 1 Danish Ramazzini Centre, Department of Occupational Medicine – University Research Clinic, Goedstrup Hospital, Herning, Denmark; 2 Department of Psychology, National Center of Psychotraumatology, University of Southern Denmark, Odense, Denmark; 3 Department of Occupational and Environmental Medicine, Odense University Hospital, Odense, Denmark; 4 Department of Clinical Research, University of Southern Denmark, Odense, Denmark

**Keywords:** Work-related violence and threats, Social support, Depression

## Abstract

**Introduction:**

Employees working at psychiatric wards are at risk for work-related threats and violence that may impact their physical and mental health. Studies have found that crisis social support may mitigate these adverse health effects.

**Purpose:**

To examine the effects crisis social support on depressive symptoms 3 months after a violent or threating work incident and furthermore, to examine the effect of variations in prolonged social support on depressive symptoms during 3 months after a violent or threating incident.

**Methodology:**

After exposure to work-related violence and threats at work, the employees received a questionnaire within the first month and after 3 months. Right after the incident, 374 employees answered both the depression and crisis support items and were included in the analyses. 3 months later 276 employees answered both the depression and social support items. Prospective associations between crisis social support and depression were calculated using stepwise regressions and linear mixed models.

**Results:**

Crisis social support at T1 was significantly and inversely associated with a lower level of depressive symptoms at T2, Std. Beta =  − 012, t (3) =  − 2.1, *p* = .040. Employees experiencing either a stable or increasing level of support from T1 to T2 had significantly lower levels of depressive symptoms compared to employees who experienced a decrease in support in the same period, mean difference_Stable–Decreasing_ = 4.0 t (190) = 5.2, *p* = 0.006 and mean difference_Increasing–Decreasing_ = 7.6, t (189) = 5.3, *p* < .001.

**Conclusion:**

The study results indicate that depressive symptoms following work-related violence or threats can be mitigated by prolonged social support. We recommend that organizations continue to offer crisis social support in the subsequent months, and not just immediately after a violent or threating incident.

## Introduction

Work-related violence and threats are a major issue in many occupations, being especially prevalent in the health care and human service sectors [1–4]. Work-related violence may be defined as “any action, incident or behavior that departs from reasonable conduct in which a person is assaulted, threatened, harmed, injured in the course of, or as a direct result of, his or her work” (p. 4) [5]. A sub category of workplace violence is the type II violence, where the perpetrator is a customer/client that has a legitimate relationship with workplace. In healthcare settings this includes patients and their families and other visitors.

Providing care to hospitalized psychiatric patients put staff in risk of work-related threats and violence as they experience high rates of verbal aggression and physical assault [6]. A study found that 82.8% and 61.6% of staff working at Danish psychiatric wards reported that they had been exposed to work-related threats or work-related violence respectively during the last year, a high level consistent with global trends [7]. Furthermore, a recent review among health care employees found that 61.9% reported being exposed to work-related violence and furthermore, 42.5% reported having been exposed to non-physical violence. Especially at psychiatric and emergency settings the frequencies of work-related violence are found to be high [2, 3].

Exposure to work-related violence or threats is often associated with mental health problems including shock, frustration, anger, irritability, depression, acute stress disorder, sleep disturbances, burnout, and increased concerns about workplace safety [8–10]. For instance, a study among mental health care staff found that 20% of psychiatric inpatient employees experienced mental health problems after exposure to work-related violence or threats [11]. The health consequences might be even worse as work-related threats and violence has been associated with posttraumatic stress disorder [8, 12, 13], burnout and anxiety [8, 9, 14]. Furthermore, work-related violence and threats may have detrimental impact on coping and cognitions of health care employees and has been associated with reduced job satisfaction, increased intention to leave, and reduced or organizational commitment [15].

Also, depression has been investigated as a consequence of work related violence and threats [16]. Studies have found that work-related violence can lead to depression for employees working in the health care [17–20]. For instance, a study among medical staff in China found that both verbal and physical violence at work was significantly associated with increased risk for depression [21] while another study among homecare employees found increased risk of sleep problems, stress and depression following work-related violence [22].

Social support refers to actual or available social resources in times of need typically to help the individual to cope with different stressors [23]. Social support may be in the form of emotional, informative or practical support from co-workers, leaders, friends and/or families. The broader social support literature has emphasized the importance of perceptions of social support to health and mental health outcomes. More specifically, reviews have found consistent evidence for the notion that social support is an important protective factor against depression [24, 25]. Following exposure to traumatic events, social support is associated with fewer somatic symptoms, greater well-being, lower likelihood of developing PTSD, and better readjustment [26, 27].

In line with these findings, a growing number of studies indicate that social support is a recommendable workplace strategy to reduce the negative impact following work-related violence and threats. For instance, a cross-sectional study among clinical employees in forensic mental hospital found that social support from both coworkers and supervisors was significantly associated with lower safety concerns, fewer physical health symptoms and fewer depressive symptoms compared to employees who did not receive social support from coworkers and supervisors, following patient assault [28]. A Canadian study among health care employees found that social support decreased the negative consequences of physical violence, vicariously experienced violence and psychological aggression on emotional well-being, somatic health and job-related affect [29]. Furthermore, studies have found that organizational social support reduce the intension to leave and moderate the negative effects of work-related violence on employee well-being, depression, job satisfaction, and organizational commitment following work-related violence or threats [30–32]. However, these studies are all cross sectional in design making the causal association difficult to determine. Finally, a qualitative study found that a phone call, a letter, or a visit to the hospital assisting the victim of work-related violence in order to obtain medical care, legal and administrative advice was experienced to be helpful [33].

As shown, social support may be associated with less health consequences after being exposed to work-related violence. This is in line with the social causation model claims that social support buffers against health consequences following exposure to work-related violence. However, the causal order may also be reversed as it is indicated by social selection model, where health consequences caused by exposure to work-related violence can reduce social support resources.

However, it is unclear at what time following the violent or threating incident the social support must be given to be beneficial. Furthermore, it is unclear how variations in social support over time might be associated with employees' well-being. Adding a temporal dimension to the models of support reduces the ambiguity found in studies which differentiate types of support, but at the same time overlook the timing of the social support [34].

Therefore, this present study will examine the association between social support and health over time following exposure to work-related threats and violence. The research questions are:

1. Are social support within the first month of exposure to work related violence or threats, associated with level of depression symptoms after 3 months?
2. Are change in level of social support in the first 3 months after exposure to work related violence and threats, associated with level of depression symptoms in the same time period?

## Methods

Every public workplace in Denmark is required to have a violence policy. A violence policy includes a definition of violence and threats and procedures to prevent and handle work-related violence and threats. It is the employers' responsibility to ensure that the employees know the violence policy of the workplace and thus the workplace's definition of work-related violence. The employee must report violent or threating incidents to the workplace's report system based on his/her subjective perception of a violent or threating incident. The employee may also ask the safety representative to report the violent and threating incident.

In collaboration with the chief top safety manager, all staff working at psychiatric wards and psychiatric outpatient wards in Region South, Denmark were potential participants in this study. The participants are employees, who reported an incidents of workplace violence or threat to the workplace's reporting system. When an incident of work-related violence or threats was reported at one of the participating psychiatric wards, the research team was informed and sent a questionnaire to the victim within the first month after registration (T1). After 3 months, the employee received a follow-up survey (T2). Sampling and data collection were completed from May 2012 to May 2015.

A total of 443 incidents of work-related violence and threats were registered throughout the sampling period. At T1, 374 individuals answered both depression and social support questions and these respondents were included in the analysis. At T2, 276 (74%) answered both depression and social support questions.

As mentioned all participants have been exposed to work-related threats or violence. In the questionnaire at T1, we asked which kind of threats or violence the employees have been exposed to leading to report the incident. Threats were measured by 6 items asking about having experienced e.g. being threatened with objects, being threatened with beatings, written threats, being scolded or shouted at in at threatening manner. Violence was measured by 11 items asking about having experienced e.g. physically violent behaviors included being hit, spat on, hit with an object, scratched/pinched, shoved, held firmly, punched with a fist, kicked, bitten, or being hit with a thrown object. The most threating incident the participants were exposed to was threating in an insulting manner and threats of beating; The most common violence incident the participants were exposed to was being shoved and being hit. For more details, see elsewhere [35].

### Questionnaires

#### Crisis social support

Was measured by the Crisis Support Scale [36, 37]. The crisis support scale is an instrument that is intended to measure received social support.

It consists of seven items, the first five items are related to positive social support, the sixth one is about feeling let down by others, and the last is about overall social support satisfaction. The positive items refer to, e.g., ‘Was there someone who would listen when you needed it’, ‘Were people sympathetic and supportive’. They are assessed on a 7-point Likert scale (1 = never to 7 = always on the first six items, and 1 = very unsatisfied to 7 = very satisfied on the last item). Scores on item 6 was reversed. All seven items were summed, rendering a scale form 7–49 according to manual. The scale has shown good reliability and validity [36] and in the present study it showed satisfactory internal consistency with Cronbach's Alpha = 0.70 at both T1 and T2. In the analysis answering the first research question, we used the sum score scale as primary explanatory variable.

In the analysis answering the second research question, we further created two variables measuring the change in crisis support from T1 to T2. Here we calculated a sum scale where the scale sum score at T1 was subtracted from the sum score at T2. This created a new sum scale from − 42 to 42 were scores below zero indicates a reduction in support and above zero an increase in support.

For the post hoc analysis we further created a categorical variable to identify three groups of change in support. Group 1 = *Decreasing support* including all respondents who reported a decrease of 10% or more from T1 to T2. Group 2 = *Stable support,* including all respondents within ± 10% change in support from T1 to T2. Group 3 = *Increasing support,* including all respondents who reported an increase in support of 10% or above, from T1 to T2. The sum scale and category scale of support change was calculated specifically for the present study to allow for an assessment of change in support.

### Outcome: depression

Level of depression was the study outcome and was measured with the Major Depression Inventory (MDI). Major Depression Inventory has been developed to measure DSM-IV and ICD-10 diagnoses of major (moderate to severe) depression by the patients’ self-reported symptoms developed by Bech and collogues [38–40]. The MDI consists of 10 items, where items 8 and 10 both have sub-items why the scale consists of a total of 12 items. Each item measures the presence of depressive symptoms during the past 2 weeks. The response categories were "All the time"(5), "Most of the time"(4), "Slightly more than half the time"(3), slightly less than half the time"(2), "Some of the time"(1), "At no time"(0). The scale was used as an index in which the ten primary items were summed resulting in a sum score scale from 0 to 50, according to the manual. The instrument has been validated in a Danish population [40], and in the present study it showed excellent internal consistency with Cronbach’s alpha at T1 = 0.96 and T2 = 0.93.

### Background information

Information about gender was retrieved from the baseline survey measured as male or female and age was measured as age in whole years at the time of the violent incident.

### Data analysis

Due to response rate of 74% at follow-up, we assessed attrition with logistic regression to detect if any of the baseline variables predicted dropout. All respondents were coded into a binary variable completer versus non-completers, based on their participation at T2. Variables measured at baseline (depression, support, gender and age) were set as predictors for the binary outcome of completion. Missing data was also assessed, with the missing values analysis tool from SPSS, and indication of data predicting missing values, was further assessed with Littles MCAR test of missing values.

Data was then visually assessed in regards to normal distribution, homoscedacity and linearity with distribution graphs, residual and PP plots. The MDI scores at both T1 and T2 were skewed towards the lowest values. To accommodate for this violation of assumptions, we used bootstrap in the regression model and the robust estimation function in the generalized mixed model. Data were deleted pairwise in the descriptive analysis and in the stepwise regression model. All data were kept in the repeated measure analysis models to secure a maximum of data points in the models.

To answer the first research question on the association between support within the first month and level of depression after 3 months, stepwise regression was used. Here, MDI sum score measured at T2 was used as outcome. At step one, age and gender was entered as explanatory variables and at step 2, level of support measured at T1 was entered. Bootstrap based on 10,000 re-samplings were used. Bootstrap allowed calculation of effect sizes and 95% confidence intervals based on the actual sample distribution, thus accommodating for the skewed distribution of the depression score. Regression analysis was done in IBM SPSS 27.0 (Armonk, NY, USA).

To answer the second research question regarding association between the change in level of support and level of depression, two sets of analysis were conducted. First, we ran a linear mixed model using the level of depressive symptoms at T1 and T2 as outcome. We used the sum score of change in support as primary explanatory variable also including age, gender, and time as predictors of depression symptoms. Further we included an interaction term of time*change in support to assess how change in support might act as moderating factor of change in depression scores from T1 to T2. The fixed effects were calculated using the Satterthwaite approximation and the robust estimation function to accommodate for the skewed outcome measure. We included a random effect, allowing for random intercept, at the level of the individual. This was to accommodate for the lack of independence between measures at T1 and T2 due to the repeated measure design. Analysis was done, using then genlinmixed function with the scaled identity covariance matrix in IBM SPSS version 27.0 (Armonk, NY, USA).

In the post hoc analyses, the categorical variable of the three groups of change in support was used as main explanatory variable. Depression sum score at T1 and T2 was set as outcome. The model also included age, gender, and time as explanatory variables. Support at T1 was entered to adjust for the effect of perceived support within the first month and finally, we included the interaction term of time*categories of change, to assess the effect of change in support on change in depression symptom over time. A random effect was included, allowing for random intercept at the level of the individual respondent to accommodate for non-independence between measures at T1 and T2. Comparisons of the estimated marginal means between groups at T1 and T2 were calculated using the genlinmixed function with robust estimations and the Satterthwaite approximation, with Bonferroni adjusted levels of significance. This model was also executed in IBM SPSS version 27.0 (Armonk, NY, USA).

Due to a large amount of missing data at T2 we chose to run sensitivity analysis based on multiple imputation datasets. Regression based estimation was used where all measures at T1 were set as predictors for imputation of depression and support at T2. In all we imputed 10 dataset and used these to first run analysis based on the regression model and secondly, the genlinmixed model based on sum scores.

## Results

Descriptive analysis showed that the sample primarily consisted of women and had a relatively high level of seniority with a mean age above 40 years. The level of depressive symptoms was generally low in the total sample. Also, the descriptive data indicated that level of support increased over time (Table 1). This was corroborated with a paired sample t-test showing a significant increase in mean level of support from T1 to T2: mean difference T1 − T2 = − 2.0, t(241) = − 4.2, *p* < 0.001.

**Table 1:** Descriptive statistics on the study sample presented as mean and standard deviation (SD) or percent % and number of respondent (N). Results are presented within the total sample and each of the groups *decreasing-*, *stable*- and *increasing* support

Variables	Total sample	Decreasing support	Stable support	Increasing support
Gender				
Male	17.9% (67)	11.5 (6)	18.4 (16)	20.4 (21)
Female	82.1 (307)	88.5 (46)	81.6 (71)	79.6 (82)
Age	41.6 (11.1)	43.8 (12.6)	40.2 (10.4)	41.3 (10.9)
Support T1	34,8 (6.5)	39.2 (5.2)	35.4 (5.5)	31.1 (6.4)
Support T2	36.6 (6.0)	31.5 (5.4)	35.3 (5.3)	39.7 (4.7)
Level of depression T1	6.0 (10.1)	7.5 (10.2)	7.5 (10.6)	3.3 (5.8)
Level of depression T2	5.4 (7.5)	8.6 (9.0)	5.9 (8.5)	3.7 (5.0)

Dropout analysis showed that none of the variables at T1 predicted attrition at T2. Missing data amounted to a total of 20.2% missing data points including data from both time points of measurement. Two items had a large percentage of missing data. Here, 37.7% of the data points were missing in the depression items at t2 and 37.6% of the data points were missing in the support items at T2. Attrition probably explained the major part of the missing data points at T2. Further, none of the variables within the dataset predicted missing values (Little MCARS, Chi Square (31) = 26.2, *p* = 0.710), which indicates that data might be missing completely at random.

The stepwise regression showed that support at T1 significantly predicted level of depression 3 months after the violent incident: R^2^ change = 0.2, F-change (1.227) = 4.2, *p* = 0.040, with an inverse association between support at T1 and depressive symptoms at T2: Std. Beta = − 0.12, t (3) = − 2.1, *p* = 0.040. The association between change in support and level of depression at T1 and T2 improved fit to data substantially when comparing the intercept-only model with the models based on change of support (Table 2).

**Table 2:** Model fit represented with the Akaikes information criteria and Bayesian information criteria of the intercept only model, the full model of sumscore change in support and the model with categories of change in support N.B. Change in BIC > 10 is a strong indicator of improved fit to data

Model	Akaikes information criterion	Bayasian information criterion
Intercept only model	4771.5	4767.0
Model with sum score of change in support	3173.0	3165.5
Model of categories of change in support	3133.2	3141.4

Overall, there was no statistically significant change in level of depression from T1 to T2. Change in support was significantly and inversely associated with the overall level of depression but not significantly associated with any change in depression (time*change in support) from T1 to T2 (Table 3).

**Table 3:** Fixed effects and parameter estimates of the full model presented with F-statistics and degrees of freedom (F(df)), model coefficients (coeff.) and 95% confidence intervals (95% CI) as well of coefficient statistics (t) and level of significance (*p*) parameter estimates and standard errors (std) test statistic (t) level of significance (*p*)

Factor	F(df)	Coeff	95% CI	t	*p*
Intercept		9.8	[6.1–13.6]	5.2	< .001
Age	5.2 (1.155)	− .0.1	[− 2.1 to 1.8]	− 0.1	.886
Gender	0.02 (1.103)	− .0.1	[− 0.2 to − 0.0]	− 2.3	.024
Time	(1.138)	0.1	[− 1.0 to 1.2]	0.1	.895
Change in support	15.0 (1.148)	− 0.2	[− 0.4 to − 0.1]	− 3.6	.000
Time*change in support	0.65 (1.151)	0.0	[− 0.1 to 0.2]	0.3	.799

Comparing the three groups of support, i.e. the group with decreasing support, the group with stable support and the group with increasing support, a pattern emerged, where the decreasing group of support showed higher level of depression at both T1 and T2 compared to the other two groups. Also, the group with increasing support had the lowest level of depression symptoms at both T1 and T2 (Table 4).

**Table 4:** Estimated means of depression in the groups of *decreasing* -, *stable-*, and *increasing support* 1 and 3 months after a violent incident presented with mean and 95% confidence intervals

	Decreasing support	95% CI	Stable support	95% CI	Increasing support	95% CI
Time 1	8.5	[5.7–11.2]	7.4	[5.1–9.7]	2.0	[0.7–3.3]
Time 2	9.9	[7.4–13.4]	5.8	[4.1–7.5]	2.3	[1.0–3.6]

Statistical comparisons of the three groups showed that only the group with increasing support had a statistically significant lower level of depression symptoms compared to the two other groups at T1. At T2, the level of depression was statistically significantly lower in the groups with stable and increasing support, and the group with increasing support still had the lowest level of depression symptoms (Table 5).

**Table 5:** Estimated mean difference of depression symptoms in in the groups of *decreasing* -, *stable-*, and *increasing* support presented across the time points T1 and T2. Presented mean difference with 95% confidence intervals (CI) as well as test statistics (degrees of freedom) and Bonferroni adjusted level of significance (*p*)

	Estimated mean difference	95% CI	Test statistics	*P*
Time 1				
Stable–decreasing	1.1	[− 2.3 to 4.5]	t(142) = 0.6	.599
Increasing–stable	5.3	[2.4–8.4]	t(142) = 4.2	.000
Increasing—decreasing	6.4	[2.7–10.2]	t(142) = 4.2	< .001
Time 2				
Stable–decreasing	4.0	[1.2–7.0]	t(190) = 5.2	.006
Increasing–stable	3.5	[1.0–6.1]	t(302) = 3.2	.004
Increasing–decreasing	7.6	[4.2–11.1]	t(189) = 5.3	< .001

t-statistic is significant at level *p* < .050

Sensitivity analysis showed the same pattern of effect sizes and level of significance as seen in the main analysis, with slightly stronger effects and significance levels (Appendix 1a and 1b).

## Discussion

This prospective study found that perceived crisis support 1 month after a violent incident was significantly associated with a lower level of depressive symptoms measured 3 months after the violent incident. The study also found that there was an inverse association between change in support and overall level of depressive symptoms throughout the 3 months' period. Finally, the study found that employees experiencing either a stable or increasing level of support in the period between 1 and 3 months after the violent incident, had significantly lower levels of depressive symptoms compared to employees who experienced a decrease of social support in the same period, even when adjusted for the level of support within the first month after the violent incident.

The results in this study are in line with studies in workplace settings that have found that social support is a protective factor against the negative health consequences following work-related threats and violence [29, 30, 32, 41]. The association between support and depression, following violence can be substantiated in several theoretical explanations. Following exposure to work-related violence, employees seek others to obtain help, support, or understanding. Positive reactions may be likely representing a confirmation of these expectation that drove victims to seek social support. Social support from others at work may thus protect employees from the pathogenic influence of work-related violence by enhancing individuals’ perceived ability to cope with the work-related violence, reducing negative appraisals of work-related violence and reducing harmful psychological responses to work-related violence. Furthermore, the experience of social support enhances a sense of meaningful shared relations and belonging to a community, an important aspect of human life that might reduce negative psychological reactions. At the same time, social support also implies a recognition of the violence incident actually took place and was significant and the victim is recognized as valuable.

One the other hand, decreasing social support or negative reactions from colleagues might interfere with natural recovery processes by leading to unhelpful trauma-related cognitions or avoidance coping [42]. One explanation for declined social support may be due to the individuals in the social surroundings who might feel uncomfortable to interact with those who were exposed to work-related violence. Over time, severity of psychological or psychiatric symptom following work-related violence and threats may undermine subsequent benefits of social support, through the affected individual’s increased tendency for withdrawal and loss of interest in interpersonal activities. Depressive states are known to cause withdrawal from social relations and ruminations coloured by self-blame and negative self-appraisal, which may contribute to interpretations that support is unavailable. That is, perceptions of low support are decreased among individuals with more depressive symptoms following work-related violence and threats. This latter pathway may be due to social selection, whereby individuals with increased psychiatric pathology are selected out of supportive social relationships, or perceive them to be less available [43]. For instance, a study with measures of social support and PTSD, 2–6 months, 5–9 months, and 14–19 months after a disaster found that persons who received less emotional support in the early aftermath of the disaster tended to have higher subsequent posttraumatic stress. However, persons with higher posttraumatic stress in the early post disaster period were more likely to subsequently report lower levels of emotional support [44]. Furthermore, earlier studies have found that exposure to violence is indirectly and positively associated with an increase in negative emotional coping [45]. In our study the fact that the group with increasing support had a lower level of depressive symptoms at T1 might indicate that this group had more resources to seek out support and profit from it. However, these possible causal mechanisms need to be corroborated in future studies on workplace violence and support.

The results of this study extend existing research in two major ways. First, previous studies finding a mitigating effect of social support on mental health following incidents of work-related threats and violence, are based on cross-sectional designs whereas this study is based on prospective data throughout a period of 3 months. Although the study did not find a statistical significant change in symptoms, which might be due to sample size, since interaction effects are high to detect in medium to low powered datasets, there was an indication that only the group experiencing a decrease in support had an increase in depressive symptoms throughout the 3 month after the violent incident. This indicates a need to uphold crisis support through longer time periods than just immediately after the incident in order to prevent mental health problems following violence, which have also been indicated in other studies regarding support after overwhelming emotional incidents [46].

Another explanation why the study did not find statistical significant changes in symptoms, may be that other components of social support are more effective than crisis social support to decrease the risk for depressive symptoms following exposure of work-related violence. Furthermore, an explanation may also be that different kind of social support influence symptoms in different types of responders (e.g. doctors, nurses).

The study found that decreased support during the first 3 months, irrespective of the level of support within the first month, were associated with a higher level of depressive symptoms. As pointed out by Leather et al. [30] effective social support is most likely a product of source, type and timing. In relation to the last dimension, timing, we included two measure points for social support. According to Jackson [34] it is useful to think about support sequences because support unfolds over time. We found that a stable or increasing level of social support was associated with lower levels of depressive symptoms compared to staff that experienced a decrease of support within the first 3 months. The study also has a bearing to the seminal Kaniasty and Norris study [47] on the social mobilization and social deterioration of social support. They focused on a total sample of disaster survivors and could demonstrate that the continuous PTSD level over time caused a decrease in social support. Therefore, by analyzing subgroups of varying social support one might find different outcomes and trajectories over time.

Practical implication of this study is that the negative health-related consequences of workplace violence should be mitigated by interventions that enhance the availability of crisis social support for employees experiencing workplace violence. Furthermore, supervisor and colleagues should be aware of the need to offer social support throughout the subsequent months following a violent and threating incident, in order to prevent the risk of development of depressive symptoms.

### Limitations

The study has several limitations. First, all participants in the study have been exposed to work-related violence or threats. This means that we cannot compare exposed employees with employees who haven't been exposed to work-related violence and threats and thus examine whether the extent of depressive symptoms was lower or higher among the exposed employees compared to those who have not been exposed to work-related violence and threats. Furthermore, we do not know if any of the employees had symptoms of depression before the violent incident. This means that we do not know whether the level of depression among the employees who had been exposed to work-related violence and who experienced low levels of social support, was lower before the violent or threatening incident.

Secondly, the was a drop-out from T1 to T2. Although attrition analysis did not find that any prediction of attrition based on the variables in the dataset, it is possible that an underlying factor caused attrition, why the study results should be interpreted with some caution regarding a possible bias. However, sensitivity analysis based on imputed datasets, showed the same pattern of results as the main analysis, just as previous studies using data collected from questionnaires, have shown that although certain characteristics were related to those who initially chose to participate and especially to those who participated at follow-up, it did not have any large influence on the relative risk estimates measured in the studies [48, 49]. Therefore, we believe that the study findings are not primarily resulting from systematic bias caused by attrition. A third issue refers to common method effects by using only self-reported data in the study [50] which may result in spurious associations between the study variables. Fourth, the data are 7–10 years old and the available social support could have changed in respect to level and type of support within the recent years. However, the crisis support scale is still a widely recognized tool for measuring support, even today, and we believe that there is no basis to assume that the basic human causal mechanisms involved in the processes regarding violence exposure, support and mental health have changed fundamentally. We believe that our data are still relevant today. Finally, the source of social support is unclear. The received social support can be received from outside the workplace as well as inside the workplace.

The study also has several strengths. First and foremost, the study is prospective in design with 2 data collections during 3 months. This means that the time dependence between variables are more reliable than if the study had been a cross-sectional design. Next, all participating psychiatric wards were recruited from the same region, which means that workplace practices, policies, and procedures to prevent violence and threats and regarding support to employees following exposure to work-related violence were similar across the participating work units. Also, the incidents were likely not minor as they were reported to the Labour Market Insurance.

## Conclusions

This study results suggest that that prolonged crisis support following work-related violence and threats mitigates the negative health effects of violence on depressive symptoms following exposure tor work-related violence and threats. The negative health consequences for employees following exposure for work-related violence and threats should be reduced by enhanced focus on the availability of crisis support for employees for a longer period of time following work-related violence and threats. Immediate social support for employees who have been exposed to work-related violence is recommended, but it is equally important for supervisors to follow up and offer social support in the following months.

In addition, the study results should provide impetus for future research on the effects of organizational support on workplace violence including both type of social support, source of social support and timing of social support.

## Data Availability

The datasets used and/or analyzed during the current study are available from the authors on reasonable request.

## Appendix 1a

Pooled estimates of regressionmodel based on 10 imputed datasets. Presented with unstandardized regression coefficient (Beta coef.) standard error (std. err.) t statistics (t) and level of significance (*p*).

**Table:**

Factor	Beta coef	Std. err	t	*p*
Constant	12.40	3.1	4.0	< 0.001
Age	− 0.10	0.0	− 1.6	0.114
Gender	0.20	1.3	0.1	0.885
Support T1	− 0.10	0.07	− 2.0	0.042

t-statistic is significant at level *p* < 0.050

## Appendix 1b

Pooled estimates of general mixed model on association between change in support and level of depression sympotoms, based on 10 imputed datasets. Presented with parameter estimates (estimate), standard error (std. err.), t statistics (t) and level of significance (*p*).

**Table:**

Factor	Estimate	Std. err	t	*p*
Intercept	25.80	2.88	9.0	< 0.001
Age	− 0.08	0.03	− 2.3	0.019
Gender (male as referent)	− 0.96	0.95	− 1.0	0.312
Change in support	− 0.50	0.06	− 7.5	< 0.001
Time	− 0.20	0.12	− 0.3	0.812
Time*change in support	0.01	0.00	0.1	0.768

t-statistic is significant at level *p* < 0.050

## References

[1] Andersen LP (2018). Work-related threats and violence in human service sectors: the importance of the psycho-social work environment examined in a multilevel prospective study. Work.

[2] Liu J (2019). Prevalence of workplace violence against healthcare workers: a systematic review and meta-analysis. Occup Environ Med.

[3] Mento C (2020). Workplace violence against healthcare professionals: a systematic review. Aggress Violent Behav.

[4] Flannery RB, Wyshak G, Flannery GJ (2018). Characteristics of international staff victims of psychiatric patient assaults: review of published findings, 2013–2017. Psychiatr Q.

[5] International Labour Office, International Council of Nurses, World Health Organization, and Public Services International (2002). Framework guidelines for addressing workplace violence in the health sector.

[6] Phillips JP (2016). Workplace violence against health care workers in the United States. N Engl J Med.

[7] Spector PE, Zhou ZE, Che XX (2014). Nurse exposure to physical and nonphysical violence, bullying, and sexual harassment: a quantitative review. Int J Nurs Stud.

[8] Nyberg A (2020). Workplace violence and health in human service industries: a systematic review of prospective and longitudinal studies. Occup Environ Med.

[9] Rudkjoebing LA (2020). Work-related exposure to violence or threats and risk of mental disorders and symptoms: a systematic review and meta-analysis. Scand J Work Environ Health.

[10] Al Ali S, Pihl-Thingvad J, Elklit A (2021). Does acute stress disorder predict posttraumatic stress disorder following workplace violence? A prospective study of psychiatric staff. Int Arch Occup Environ Health.

[11] van Leeuwen ME, Harte JM (2017). Violence against mental health care professionals: prevalence, nature and consequences. J Forensic Psychiatry Psychol.

[12] Andersen LP (2019). Work-related threats and violence and post-traumatic symptoms in four high-risk occupations: short-and long-term symptoms. Int Arch Occup Environ Health.

[13] Pihl-Thingvad J (2019). Are frequency and severity of workplace violence etiologic factors of posttraumatic stress disorder? A 1-year prospective study of 1,763 social educators. J Occup Health Psychol.

[14] Lanctôt N, Guay S (2014). The aftermath of workplace violence among healthcare workers: a systematic literature review of the consequences. Aggress Violent Behav.

[15] Madlock PE, Kennedy-Lightsey C (2010). The effects of supervisors’ verbal aggressiveness and mentoring on their subordinates. J Bus Commun (1973).

[16] Andersen LPS (2019). Depressive symptoms following work-related violence and threats and the modifying effect of organizational justice, social support, and safety perceptions. J Interpers Violence.

[17] Fang H (2018). Depressive symptoms and workplace-violence-related risk factors among otorhinolaryngology nurses and physicians in Northern China: a cross-sectional study. BMJ Open.

[18] Tong C (2019). The effect of workplace violence on depressive symptoms and the mediating role of psychological capital in Chinese township general practitioners and nurses: a cross-sectional study. Psychiatry Investig.

[19] Sui G (2019). Associations of workplace violence and psychological capital with depressive symptoms and burn-out among doctors in Liaoning, China: a cross-sectional study. BMJ Open.

[20] Li X, Wu H (2021). Does psychological capital mediate between workplace violence and depressive symptoms among doctors and nurses in Chinese general hospitals?. Psychol Res Behav Manag.

[21] Wang H, Zhang Y, Sun L (2020). The effect of workplace violence on depression among medical staff in China: the mediating role of interpersonal distrust. Int Arch Occup Environ Health.

[22] Hanson GC (2015). Workplace violence against homecare workers and its relationship with workers health outcomes: a cross-sectional study. BMC Public Health.

[23] Hobfoll SE, Stokes JP, Duck S, Hay DF, Hobfoll SE, Ickes W, Montgomery BM (1988). The process and mechanics of social support. Handbook of personal relationships: theory, research and interventions.

[24] Gariepy G, Honkaniemi H, Quesnel-Vallee A (2016). Social support and protection from depression: systematic review of current findings in Western countries. Br J Psychiatry.

[25] Rueger SY (2016). A meta-analytic review of the association between perceived social support and depression in childhood and adolescence. Psychol Bull.

[26] Wang Y (2021). Social support and posttraumatic stress disorder: a meta-analysis of longitudinal studies. Clin Psychol Rev.

[27] Wright BK (2013). Support mechanisms and vulnerabilities in relation to PTSD in veterans of the Gulf War, Iraq War, and Afghanistan deployments: a systematic review. J Trauma Stress.

[28] Kelly EL (2017). Sources of social support after patient assault as related to staff well-being. J Interpers Violence.

[29] Schat ACH, Kelloway EK (2003). Reducing the adverse consequences of workplace aggression and violence: the buffering effects of organizational support. J Occup Health Psychol.

[30] Leather P (1998). Exposure to occupational violence and the buffering effects of intra-organizational support. Work Stress.

[31] Courcy F, Morin AJ, Madore I (2019). The effects of exposure to psychological violence in the workplace on commitment and turnover intentions: the moderating role of social support and role stressors. J Interpers Violence.

[32] Liu L (2013). Positive resources for combating depressive symptoms among Chinese male correctional officers: perceived organizational support and psychological capital. BMC Psychiatry.

[33] De Puy J (2015). Clinically assessed consequences of workplace physical violence. Int Arch Occup Environ Health.

[34] Jackson LA (1996). Achieving positive social identity: social mobility, social creativity, and permeability of group boundaries. J Pers Soc Psychol.

[35] Andersen LP, Elklit A, Pihl-Thingvad J (2021). Work-related violence and organizational commitment among health care workers: does supervisor’s support make a difference?. Int Arch Occup Environ Health.

[36] Joseph S (1992). Crisis support and psychiatric symptomatology in adult survivors of the Jupiter cruise ship disaster. Br J Clin Psychol.

[37] Elklit A, Pedersen SS, Jind L (2001). The crisis support scale: psychometric qualities and further validation. Personality Individ Differ.

[38] Bech P (1997). Quality of life instruments in depression. Eur Psychiatry.

[39] Bech P, Wermuth L (1998). Applicability and validity of the Major Depression Inventory in patients with Parkinson's disease. Nord J Psychiatry.

[40] Bech P (2001). The sensitivity and specificity of the Major Depression Inventory, using the present state examination as the index of diagnostic validity. J Affect Disord.

[41] Azar M (2016). Does administrative support negate the consequences of nurse abuse?. J Nurs Manag.

[42] Dworkin ER, Brill CD, Ullman SE (2019). Social reactions to disclosure of interpersonal violence and psychopathology: a systematic review and meta-analysis. Clin Psychol Rev.

[43] Dohrenwend BP (2000). The role of adversity and stress in psychopathology: some evidence and its implications for theory and research. J Health Soc Behav.

[44] Platt JM (2016). A longitudinal study of the bidirectional relationship between social support and posttraumatic stress following a natural disaster. J Trauma Stress.

[45] Pihl-Thingvad J (2019). Occupational violence and PTSD-symptoms: a prospective study on the indirect effects of violence through time pressure and nontraumatic strains in the occupational context. J Occup Environ Med.

[46] Taylor SE, Friedman HS (1988). Social support: a review. The Oxford handbook of health psychology.

[47] Kaniasty K, Norris FH (2008). Longitudinal linkages between perceived social support and posttraumatic stress symptoms: sequential roles of social causation and social selection. J Trauma Stress.

[48] Nohr EA (2006). Does low participation in cohort studies induce bias?. Epidemiology.

[49] Winding TN (2014). Initial non-participation and loss to follow-up in a Danish youth cohort: implications for relative risk estimates. J Epidemiol Community Health.

[50] Podsakoff PM (2003). Common method biases in behavioral research: a critical review of the literature and recommended remedies. J Appl Psychol.

